# Editorial: Global perioperative care in Africa

**DOI:** 10.3389/fmed.2025.1756735

**Published:** 2026-01-07

**Authors:** Promise Ariyo, Eric V. Jackson, Serena Cruz, Ifeoma Ekwere, John B. Sampson

**Affiliations:** 1 Department of Anesthesiology and Critical Care Medicine, Johns Hopkins University, Baltimore, MD, United States; 2 Institute of Global Perioperative Care, Harve De Grace, MD, United States; 3 Nuffield Department of Women's and Reproductive Health, University of Oxford, Oxford, United Kingdom; 4 University of Benin, Benin City, Edo, Nigeria

**Keywords:** perioperative care, critical care, research, Africa, global health

Perioperative care and critical care medicine are fundamental to providing universal health coverage, yet they remain among the least equitably distributed services worldwide. Today, an estimated 5 billion people lack access to safe, affordable surgical and anesthesia care, underscoring the urgency of system-wide solutions (1). The *Frontiers in Medicine* Research Topic *Global Perioperative Care in Africa* brings together a multidisciplinary body of evidence that underscores both the complexity and the promise of advancing surgical, anesthesia, and critical care capacity across low- and middle-income countries. These studies reflect a shared goal: using clinical innovation and research to strengthen health systems and create equity in Africa.

## Innovation grounded in context

The Research Topic opens with work that exemplifies the adaptive ingenuity of clinicians and engineers who must respond to resource constraints. In “*A comparative analysis of intravenous infusion methods for low-resource environments*,” Tomobi et al. evaluate pragmatic infusion strategies suitable for hospitals where electricity and consumables are limited. Their findings remind us that technology design must begin with end-user realities. Similarly, in “*Bridging the mismatch: observing the introduction of new anesthesia technology for a low-resource environment*,” Sampson et al. demonstrate that sustainable adoption of biomedical innovation requires participatory learning, co-creation, and local ownership. Both studies highlight that scientific rigor and contextual empathy are not opposing values but complementary foundations of sustainable innovation.

## Workforce and systems as catalysts for change

Strengthening human resources and institutional processes emerges as a unifying theme across the Research Topic. In “*Strengthening nursing knowledge and skills in perioperative cleft care: a focused training approach in Nigeria's surgical healthcare plan*,” Lawal et al. present a model for targeted, context-specific nurse education that extends beyond skill acquisition to empowerment and retention. “*The landscape of perioperative nursing education in Africa: a scoping review*” Wong et al. broadens this conversation, mapping curricular gaps and policy opportunities to professionalize perioperative nursing across the continent.

At the systems level, “*Development, implementation, and evaluation of a rapid response system at a Nigerian teaching hospital, a novel idea in sub-Saharan Africa*,” Ariyo et al. introduces an evidence-based framework for in-hospital emergency response—a novel concept in many sub-Saharan contexts. This intervention demonstrates how structured communication, early warning tools, and multidisciplinary teamwork can reduce preventable deaths and foster a culture of safety.

## Local evidence for global relevance

Building a global science of perioperative care requires evidence generated within the regions where needs are greatest. In “*Mortality and its associated factors among mechanically ventilated adult patients in the intensive care units of referral hospitals in Northwest Amhara, Ethiopia*” Tadesse et al., outcome data collected from local intensive care units illuminate risk factors that global datasets often overlook. “*Healthcare providers' knowledge, attitude, and practice toward cervical cancer screening in sub-Saharan Africa: systematic review and meta-analysis*” Delie et al. similarly emphasizes the translational value of regional research for broader health-system planning. Finally, “*Ultrasound assessment of diaphragmatic dysfunction in non-critically ill patients: relevant indicators and update*,” Yao et al. illustrates how advances in diagnostic science can inform perioperative and critical care pathways across settings.

Collectively, these articles challenge the historic one-way flow of scientific knowledge from high- to low-income contexts. By prioritizing African data, they expand the global evidence base, making it both more representative and more actionable.

## Financing, partnerships, and the path to equity

Scientific progress alone cannot sustain system transformation without corresponding investment and policy alignment. Financing mechanisms for perioperative and critical care remain fragmented, often dependent on short-term donor cycles rather than long-term infrastructure and workforce development. To realize the vision articulated in this Research Topic, health-system strengthening must be recognized as a global public good—worthy of stable financing akin to vaccination or disease surveillance programs. Without sustained investment, even the most promising innovations cannot close the surgical access gap highlighted by the Lancet Commissions's findings.

Advocacy networks such as the G4 Alliance, the Johns Hopkins Global Alliance of Perioperative Professionals, and other regional and diaspora-led coalitions have a crucial role in aligning governments, academia, and philanthropy around this agenda. Their combined advocacy underscores that equitable surgical and critical care is not a luxury of wealthy nations but a prerequisite for resilient health systems everywhere.

## Conclusion: toward a science of systems

The articles gathered under *Global Perioperative Care in Africa* collectively redefine what constitutes global health research. They move the field from isolated interventions toward a science of systems—where engineering, clinical medicine, public health, and economics converge to strengthen institutional capacity. This Research Topic invites continued collaboration among clinicians, researchers, engineers, and policymakers to expand the frontiers of equitable care. By embedding innovation within local systems, nurturing regional research capacity, and mobilizing global partnerships, the contributors to this Research Topic chart a path toward sustainable, evidence-based perioperative and critical care for all.

*Global health equity will be achieved not by technology alone, but by the deliberate alignment of science, solidarity, and sustained investment*.
